# Investigation of Mechanical, Thermal and Microstructural Properties of Waste Micro-Nano Nichrome (NiCr 80/20) Powder-Reinforced Composites with Polyamide 66 (PA66) Matrix

**DOI:** 10.3390/polym17202753

**Published:** 2025-10-15

**Authors:** Mehmet Ceviz

**Affiliations:** Renewable Energy Technician Program, Department of Motor Vehicles and Transportation Technologies, Keşan Vocational College, Trakya University, 22100 Keşan, Edirne, Türkiye; mehmetceviz@trakya.edu.tr

**Keywords:** PA66 composites, NiCr 80/20, waste recycling, thermal conductivity, mechanical properties, microstructure

## Abstract

This study investigates the mechanical, thermal, electrical, and microstructural properties of polyamide 66 (PA66) composites reinforced with waste-derived micro–nano NiCr (80/20) powders. Composites containing 2, 5, and 8 wt% NiCr were prepared using thermokinetic mixing and compression molding, followed by characterization via tensile testing, Shore D hardness, Fourier transform infrared spectroscopy (FTIR), differential scanning calorimetry (DSC), scanning electron microscopy (SEM) with energy-dispersive X-ray spectroscopy (EDS), and thermal/electrical conductivity measurements. Results showed a progressive increase in tensile modulus, tensile strength, hardness, and thermal conductivity with increasing NiCr content, reaching maximum values at 8 wt% filler. However, elongation at break decreased, indicating reduced ductility due to restricted polymer chain mobility. DSC and FTIR analyses revealed that low NiCr loadings promoted nucleation and crystallinity, while higher contents disrupted crystalline domains. Electrical conductivity exhibited a slight upward trend, remaining sub-percolative up to 8 wt% NiCr; conductivity modulation is modest at high filler loadings. SEM–EDS confirmed uniform dispersion at low–moderate contents and agglomeration at higher levels. The use of industrial waste NiCr powder not only enhanced material performance but also contributed to sustainable materials engineering by valorizing by-products from the coatings industry. These findings suggest that NiCr/PA66 composites have potential applications in automotive, electronics, and thermal management systems requiring improved mechanical rigidity and heat dissipation.

## 1. Introduction

Polyamide 66 (PA66), commonly known as Nylon 66, is a widely utilized thermoplastic polymer known for its exceptional mechanical properties, thermal stability, and chemical resistance [1]. It has found applications in a broad range of industries, including automotive, electronics, textiles, and consumer goods, due to its strong and resilient nature [2]. PA66 is particularly valued for its excellent mechanical properties such as high stiffness, tensile strength, and wear resistance, as well as its good thermal stability and dimensional stability at elevated temperatures [3,4,5]. However, despite its outstanding characteristics, pure PA66 suffers from inherent limitations, particularly regarding its impact strength, toughness, and resistance to high thermal stresses under certain conditions [6]. These limitations often restrict its performance in more demanding applications, especially those involving mechanical loads or exposure to harsh environments.

To overcome these limitations and unlock the full potential of PA66, extensive research has been conducted to develop PA66-based composite materials [7,8,9]. The idea of reinforcing PA66 with various fillers, including natural fibers (e.g., flax, hemp, and jute), glass fibers [10], and carbon fibers, has been widely explored [11,12,13]. These reinforcement materials help address the shortcomings of pure PA66 by improving its mechanical properties, toughness, and thermal stability [14]. Glass fiber, for example, has been found to increase the strength-to-weight ratio of PA66 composites significantly, while carbon fibers have been shown to enhance both strength and stiffness even further [15,16]. On the other hand, natural fibers offer eco-friendly alternatives, providing a renewable and biodegradable reinforcement option [17,18].

One of the promising materials for enhancing PA66′s properties is the incorporation of metal-based reinforcements, such as NiCr 80/20 powder. NiCr 80/20, an alloy consisting of 80% nickel and 20% chromium, is known for its superior corrosion resistance, high melting point, excellent thermal stability, and good mechanical strength [19,20]. These properties make NiCr 80/20 particularly suitable for high-temperature and corrosive environments, such as those encountered in the aerospace, chemical processing, and automotive industries. The alloy’s excellent resistance to oxidation and carburization, combined with its high thermal conductivity, makes it an ideal candidate for enhancing the thermal properties and overall performance of polymer-based composites [21]. Furthermore, the introduction of micro-nano-sized NiCr 80/20 powder offers a unique advantage, as smaller particles can be uniformly dispersed within the polymer matrix, leading to enhanced mechanical interlocking and improved load-bearing capacity [22].

NiCr 80/20 powder, with its superior properties such as high-temperature resistance, corrosion, and oxidation resistance, is widely used across various industrial applications [23,24]. This alloy is commonly employed in the production of heating elements, furnace components, and thermal processing devices that are exposed to high heat environments [25,26]. Additionally, it can be used in the aerospace and space industries, chemical processing, and petrochemical sectors, where components need to withstand harsh conditions. NiCr 80/20 stands out not only for its high-temperature durability but also for its excellent mechanical strength and high melting point, making it ideal for manufacturing heat exchangers, furnace parts, and other high-temperature equipment [27]. The powder is also well-suited for applications requiring long-term performance under extreme thermal conditions, making it essential material in industrial sectors demanding reliable and durable components.

Another important consideration is the increasing demand for sustainable materials [28]. The utilization of industrial waste, such as NiCr 80/20 powder comes from the coatings industry [29], provides a novel approach to recycling materials that would otherwise contribute to environmental pollution [30]. The use of waste materials not only supports sustainability but also adds an economic value to industrial products [31]. By incorporating waste NiCr 80/20 powder into the PA66 matrix, this study aims to contribute to the development of environmentally friendly and high-performance composite materials.

In recent years, there have been a growing amount of research dedicated to PA66-based composites reinforced with metallic powders, particularly focusing on improving their thermal, mechanical, and electrical properties. Beyond fiber and carbonaceous fillers, metallic powders (e.g., Cu, Ni-based alloys) have been explored to stiffen PA66 and improve heat transport, typically yielding higher modulus/hardness and modest thermal-conductivity gains, but often at the cost of ductility due to particle agglomeration and limited interfacial compatibility. Electrical percolation with spherical/near-spherical metal particles generally requires comparatively high loadings, so low-to-moderate micro/nanoparticle contents remain electrically insulating. While NiCr is widely used in high-temperature/coating applications for its oxidation resistance and stable thermophysical behavior, reports on NiCr(80/20)-PA66 bulk composites are limited, motivating the present work on recycled NiCr powders in PA66 [15,16,19,23,26,29,31]. However, despite the significant progress made in this area, there remains a gap in understanding the behavior of PA66 composites reinforced with micro-nano-sized NiCr 80/20 powders, especially when sourced from industrial waste. The goal of this research is to investigate the microstructural, mechanical, and thermal properties of PA66 reinforced with varying amounts of micro-nano-sized NiCr 80/20 powder (ranging from 2 wt% to 8 wt%), to better understand the impact of NiCr reinforcement on the composite’s overall performance.

The main objectives of this study include: (i) evaluating the mechanical properties (tensile strength and hardness) of the PA66-NiCr 80/20 composites, (ii) assessing their thermal behavior through differential scanning calorimetry (DSC), (iii) investigating the microstructure and dispersion of NiCr powder within the PA66 matrix using scanning electron microscopy (SEM), (iv) exploring the potential for recycling industrial waste by incorporating NiCr 80/20 powder into the polymer matrix, (v) analyzing the thermal conductivity of the composites to understand the impact of NiCr 80/20 on heat transfer properties, (vi) investigating the electrical conductivity of the composites to explore their potential use in electrical and electronic applications, and (vii) examining the chemical interactions and structural modifications of PA66 and NiCr 80/20 through Fourier transform infrared spectroscopy (FTIR). Through these investigations, this study seeks to provide valuable insights into the effects of NiCr reinforcement on PA66 composites and explore the potential applications of these materials in industries where high thermal conductivity, enhanced strength, and environmental sustainability are paramount.

The results of this study could significantly contribute to the development of new PA66-based composite materials with improved performance and sustainability. By demonstrating that waste NiCr 80/20 powder can be effectively used as a reinforcing agent in PA66 composites, this research aims to search possibilities for the reuse of industrial waste, offering an innovative solution to both material enhancement and waste management.

## 2. Materials and Methods

NiCr nanoparticles and microparticles were mixed with PA66 for 60 s in a thermokinetic mixer at a rotational frequency of 5250 rpm [32]. A short mixing time was selected to minimize thermal degradation of PA66. Homogeneity was ensured by the high shear forces in the thermokinetic mixer, and further confirmed by SEM–EDS uniform dispersion at 2 and 5 wt% NiCr. The NiCr nano- and microparticle content ranged from 2 to 8 wt% of the composition. Pure (Tecomid NA40 NL E, Eurotec Engineering Plastics, Kocaeli, Türkiye) (density: 1.14 g/cm^3^, melting temperature: 262 °C) was used in this study. NiCr 80/20 micro and nano particles were produced by collecting waste powders from the coatings industry (local supplier, Kocaeli, Türkiye). Firstly, the PA66 granules were dried in oven at 60 °C for 2 h to remove residual moisture. Thin plates (150 mm × 160 mm × 4 mm) were obtained from the granules using a compression molding machine (Carver Inc., Wabash, IN, USA) at approximately 270 °C under a pressure of 3 tons for 4 mm thick plate specimens [33]. The shapes and dimensions of the specimens were determined according to the test requirements and cut from the plates. The investigation of the thermal behavior of PA66–NiCr composites was carried out by Differential Scanning Calorimetry (DSC) (Mettler Toledo DSC 1, Mettler-Toledo AG, Greifensee, Switzerland). There have been uses of mass samples ranging from 5 to 10 mg. The following explains the operational protocol: Under a nitrogen environment, the samples were heated at room temperature to 300 °C at a heating rate of 20 °C/min [34]. The degree of crystallinity (X_c_) was calculated from the DSC melting enthalpy (ΔH_m_) according to

(1) $$X_{c}=\frac{{\Delta H}_{m}}{{\Delta H{}^{\circ}}_{m}(1-\phi )}$$

where ΔH°_m_ = 188 J/g is the enthalpy of 100% crystalline PA66, and ϕ is the NiCr mass fraction. FTIR measurements were carried out using a Mettler Toledo FTIR Spectrometer (Mettler-Toledo AG, Greifensee, Switzerland). The PA66 samples were cut into pieces and then clamped onto the sample stage of the FTIR spectrometer [35]. The PA66/NiCr 80/20 composites and NiCr 80/20 powder were characterized on potassium bromide (KBr) pellets. The data were recorded in the wave number range from 500 cm^−1^ to 4000 cm^−1^. The tensile test is conducted in accordance with ASTM D638-00 [36] at room temperature, using tensile test stroke control and a tensile rate of 1 mm/min. The level of toughness possessed by the composite material was evaluated using the Shore-D hardness scale. The test standard ASTM D 2240 [37] was appropriate to use. Type-D Durometer (Mitutoyo, Kanagawa, Japan); sample thickness 4 mm (compression-molded plates), ≥20 indentations/specimen at spatially separated points; 1 s approach + 1 s dwell per reading; laboratory ambient 23 ± 2 °C. The reported value is the mean ± SD across three specimens. In order to obtain an overall picture of the composite, the Durometer was pressed into it in a number of distinct locations (a minimum of 20 indentation sites per specimen). The topographic mode of a Zeiss Evo LS10 instrument was used to make the observations, which were then applied to the surfaces of samples that had been rendered conductive by metallization Au deposit. Specimens were examined on their as-molded surfaces. Prior to imaging, samples were sputter-coated with ~5 nm Au to ensure conductivity; SEM was performed (Zeiss EVO LS10, Carl Zeiss AG, Oberkochen, Germany) in secondary-electron/topographic mode at 20 kV (working distance 8–10 mm). EDS maps were collected on the same surfaces to verify Ni/Cr distribution. Because there is a potential for the samples to be damaged in any way, the electron acceleration voltage has been maintained at 20 kV.

**Figure 1:**
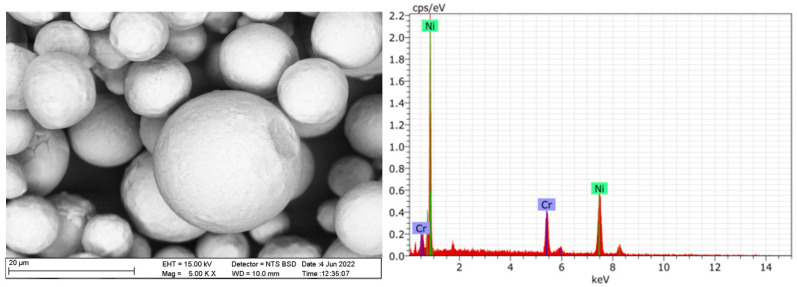
SEM image and EDS analysis of spherical NiCr powder particles.

Figure 1 presents the microstructural morphology and elemental composition of the NiCr powder. The SEM image on the left shows that the particles exhibit a predominantly spherical morphology with smooth surfaces, which is typical of powders produced via gas atomization or similar controlled processes. This morphology enhances flowability, uniform dispersion, and sintering behavior, making the powder suitable for composite reinforcement.

The EDS spectrum on the right confirms the elemental composition of the powder. Distinct peaks corresponding to nickel (Ni) are observed at approximately 0.8 keV and 7.5 keV, while chromium (Cr) peaks appear around 0.5 keV and 5.4 keV. The prominent intensity of these peaks indicates that the powder is rich in Ni and Cr, with no detectable impurities.

This analysis verifies the chemical purity and structural integrity of the NiCr powder, ensuring its effectiveness as a reinforcing phase in PA66-based composite systems.

## 3. Results

The experimental findings for the mechanical, thermal, electrical, and microstructural characterization of neat PA66 and PA66/NiCr composites are presented in this section. The results are organized to illustrate the influence of NiCr content on key performance parameters, enabling a comprehensive evaluation of the structure–property relationships. Mechanical properties, including tensile strength, modulus, and elongation at break, were assessed to determine the reinforcing effect of the metallic filler. Thermal and electrical conductivity measurements were performed to evaluate functional enhancements, while FTIR and DSC analyses provided insight into molecular interactions and crystallinity changes induced by NiCr incorporation. Additionally, Shore D hardness testing was conducted to assess surface rigidity, and SEM–EDS analyses were used to examine filler dispersion and elemental distribution within the matrix. The combined results offer a detailed understanding of how varying NiCr loadings affect the overall performance and potential applications of the composites.

**Figure 2:**
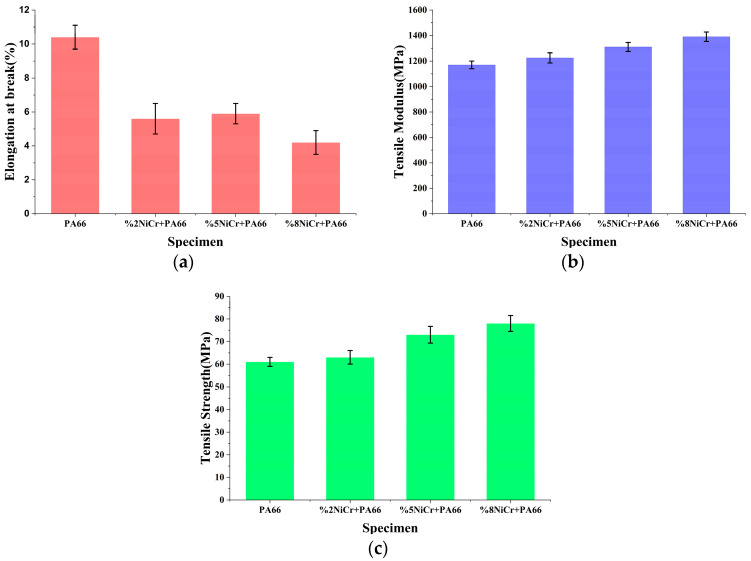
Mechanical properties of neat PA66 and PA66/NiCr composites: (**a**) Elongation at break, (**b**) Tensile modulus, (**c**) Tensile strength.

The mechanical properties of neat PA66 and PA66/NiCr composites are presented in Figure 2, where (a) shows elongation at break, (b) tensile modulus, and (c) tensile strength.

Elongation at break values (Figure 2a) decreased notably with the incorporation of NiCr particles. Neat PA66 exhibited the highest elongation (~10.3%), which dropped sharply to ~5.5% at 2 wt% NiCr. A slight recovery was observed at 5 wt% NiCr (~5.8%), while the 8 wt% NiCr composite showed the lowest elongation (~4.1%). This reduction in ductility indicates that NiCr particles restrict polymer chain mobility and promote more brittle fracture behavior.

Tensile modulus results (Figure 2b) revealed a progressive increase with NiCr loading. The modulus increased from ~1170 MPa for neat PA66 to ~1220 MPa (2 wt%), ~1300 MPa (5 wt%), and ~1390 MPa (8 wt%). This improvement is attributed to the high stiffness of NiCr particles and efficient load transfer through the filler–matrix interface.

Similarly, tensile strength (Figure 2c) exhibited a positive correlation with NiCr content, rising from ~60.9 MPa in neat PA66 to ~62.5 MPa (2 wt%), ~72.4 MPa (5 wt%), and ~77.8 MPa (8 wt%). The enhancement in tensile strength is associated with improved stress distribution and the reinforcing effect of metallic particles within the polymer matrix.

Overall, the addition of NiCr significantly improved stiffness and tensile strength while reducing elongation at break, with the best strength and modulus obtained at 8 wt% NiCr, albeit with the lowest ductility.

**Figure 3:**
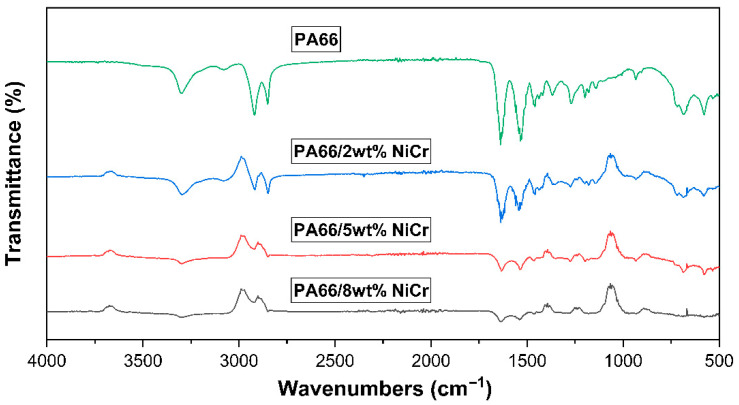
FTIR spectra of neat PA66 and PA66/NiCr composites with varying filler contents.

FTIR (Fourier Transform Infrared Spectroscopy) analysis was performed to evaluate the chemical structures of neat PA66 and PA66-based composites containing varying weight percentages (2 wt%, 5 wt%, and 8 wt%) of NiCr micro/nano powders. The spectra presented in Figure 3 clearly reveals that the molecular interactions and structural modifications induced by the incorporation of NiCr particles into the polymer matrix.

In the spectrum of neat PA66, a broad absorption band around 3300 cm^−1^ is attributed to N–H stretching vibrations, while the absorption near 2900 cm^−1^ corresponds to aliphatic C–H stretching. The characteristic amide I (C=O stretching) and amide II (N–H bending) bands are observed at approximately 1630 cm^−1^ and 1540 cm^−1^, respectively. Moreover, the peaks located within the 1200–1000 cm^−1^ range are associated with C–N stretching vibrations. These features collectively confirm the presence of the polyamide backbone and its semi-crystalline structure.

Upon the addition of NiCr powders, notable changes are observed in the FTIR spectra. For the PA66/2 wt% NiCr composite, compared to neat PA66, the broad N–H stretching band near ~3300 cm^−1^ shows a small wavenumber shift, while the amide I (C=O) band at ~1630 cm^−1^ exhibits a reduction in intensity; the amide II band (~1540 cm^−1^) and C–N stretching (1200–1000 cm^−1^) also attenuate gradually with increasing NiCr content. In the PA66/5 wt% NiCr sample, further attenuation of peak intensities and slight shifts in wavenumbers suggest enhanced interaction, likely due to increased surface contact and dispersion of NiCr within the matrix, which may restrict polymer chain mobility. In the PA66/8 wt% NiCr composite, a significant reduction in peak intensities, along with peak broadening and overlap, is observed. This behavior suggests that a higher loading of NiCr disrupts the crystalline domains of PA66, leading to increased amorphous character and altered molecular organization.

Overall, the FTIR results indicate that the incorporation of NiCr micro/nano powders into the PA66 matrix induces both physical and weak chemical interactions, with increasing NiCr content leading to progressive alterations in the polymer’s molecular structure. These findings imply that NiCr loading may significantly influence the composite’s thermal, mechanical, and surface properties, and provide a basic understanding of structure–property relationships in such hybrid materials.

**Figure 4:**
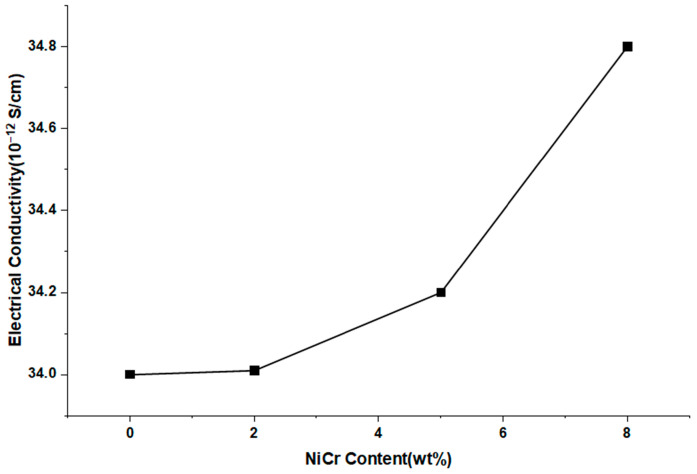
Electrical conductivity of neat PA66 and PA66/NiCr composites at different filler loadings.

The electrical conductivity of neat PA66 and NiCr-reinforced PA66 composites was evaluated to investigate the influence of conductive filler addition on the electrical behavior of the polymer matrix. As illustrated in Figure 4, neat PA66 exhibits a very low electrical conductivity, in the order of 34.0 × 10^−12^ S/cm, consistent with its insulating nature. Electrical conductivity shows a measurable but minor increase with NiCr loading; absolute values remain within the insulating range.

Upon the addition of NiCr micro/nano particles, a gradual increase in electrical conductivity is observed. Specifically, at 2 wt% NiCr, the conductivity remains relatively unchanged, indicating that the filler content is below the percolation threshold, and thus, no continuous conductive pathways are formed within the matrix. However, at 5 wt% NiCr, a slight but noticeable increase in conductivity suggests the onset of filler networking, where conductive pathways begin to emerge due to closer proximity and partial contact between the metallic particles.

At 8 wt% NiCr, conductivity rises from ~3.4 × 10^−11^ to ~3.48 × 10^−11^ S/m (i.e., 34.0 × 10^−12^ to 34.8 × 10^−12^ S/cm), which, despite being measurable, does not constitute electrical percolation. This behavior aligns with reports that spherical/near-spherical metal micro/nanoparticles in PA66 typically require substantially higher volume fractions—or high-aspect-ratio/hybrid conductive networks—to trigger order-of-magnitude jumps in σ. Accordingly, we refrain from percolation claims at ≤8 wt% and discuss antistatic/EMI implications conservatively [15,19].

The observed trend highlights the role of NiCr content in modifying the electrical properties of PA66-based composites. While the absolute conductivity values remain within the insulating regime, the increasing trend suggests the potential for tailoring electrical properties through controlled filler loading. This behavior aligns with the typical percolation theory, wherein a critical concentration of conductive filler is required to achieve a sharp rise in electrical conductivity.

**Figure 5:**
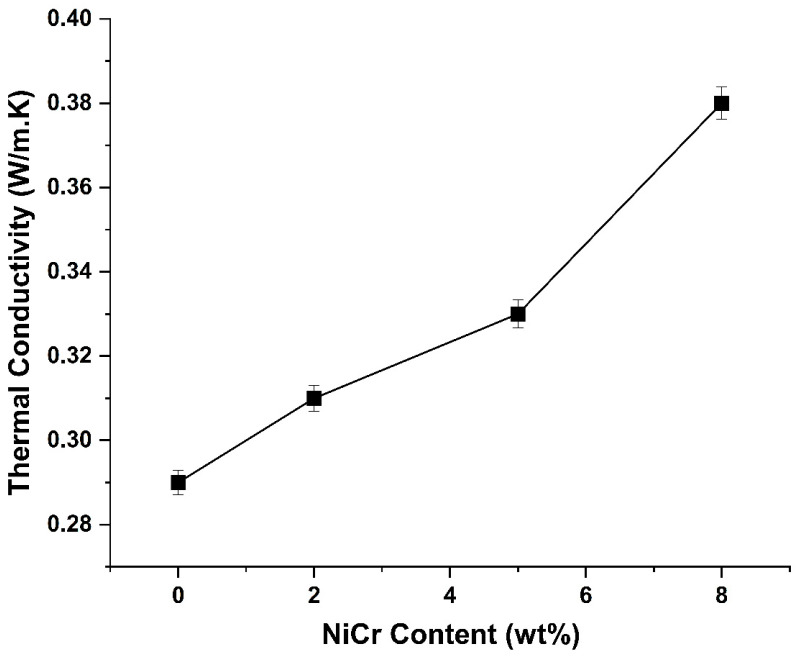
Thermal conductivity of neat PA66 and PA66/NiCr composites at different filler loadings.

The thermal conductivity of neat PA66 and NiCr-reinforced PA66 composites was measured to assess the influence of conductive filler content on heat transfer performance. As illustrated in Figure 5, an evident increase in thermal conductivity is observed with the progressive addition of NiCr particles.

Neat PA66 exhibits a low thermal conductivity of approximately 0.29 W/m·K, consistent with the inherently insulating nature of polymer materials. Upon the incorporation of 2 wt% NiCr, the conductivity increases to 0.31 W/m·K, suggesting the initial formation of a thermally conductive network within the matrix. With 5 wt% NiCr, this value further increases to 0.33 W/m·K, and reaches approximately 0.38 W/m·K at 8 wt% NiCr.

The improvement in thermal conductivity is attributed to the high intrinsic thermal conductivity of NiCr, which facilitates enhanced phonon or electron transport through the composite. Additionally, the increasing filler content likely leads to improved particle-to-particle contact and more efficient thermal transfer across the filler–matrix interface.

These findings demonstrate that the addition of NiCr significantly enhances the thermal management capabilities of PA66-based composites, making them suitable for thermal interface materials, heat dissipation components, and electronic packaging applications where improved heat conduction is essential.

**Figure 6:**
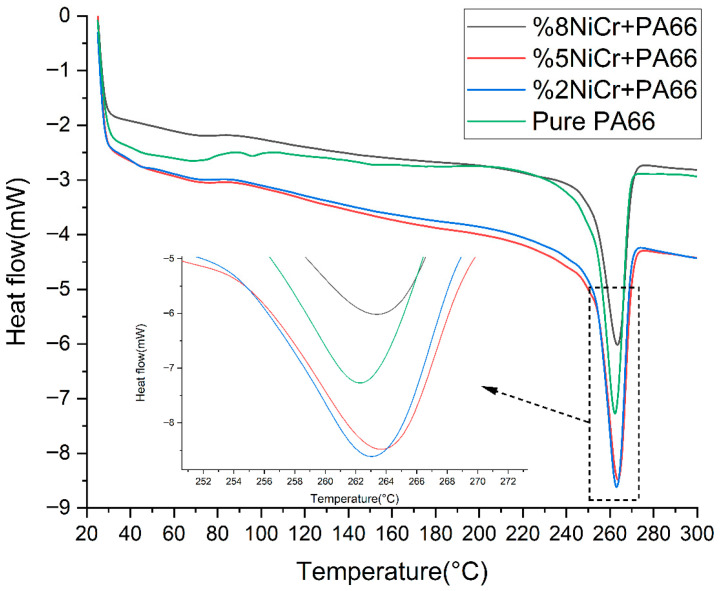
DSC thermograms of neat PA66 and PA66/NiCr composites with different filler loadings.

Differential Scanning Calorimetry (DSC) analysis was performed to evaluate the thermal transitions and melting behavior of neat PA66 and NiCr-reinforced PA66 composites with varying filler contents (2 wt%, 5 wt%, and 8 wt% NiCr). The resulting thermograms are presented in Figure 6, which reveal distinct differences in heat flow behavior as a function of NiCr content.

Calculated crystallinity values are summarized in Table 1. X_c_ increased slightly at 2 wt% NiCr, suggesting nucleation, but decreased again at 8 wt% due to agglomeration and chain restriction.

Neat PA66 exhibits a sharp endothermic peak at approximately 263 °C, corresponding to its melting temperature (Tm). This peak is characteristic of the crystalline phase of PA66 and reflects the energy absorbed during the melting of ordered polymer domains.

Upon the addition of 2 wt% NiCr, a slight shift of the melting peak towards higher temperature (~264 °C) is observed, along with an increase in peak intensity. This suggests that low levels of NiCr may promote nucleation and facilitate crystalline phase formation, likely due to enhanced thermal conductivity and localized ordering effects. At 5 wt% NiCr, the peak remains sharp but slightly broadens, indicating a balance between increased nucleation and restricted molecular mobility due to particle–polymer interactions.

In the case of 8 wt% NiCr, the melting peak appears slightly shifted and broadened, with a reduction in peak intensity. This behavior implies that excessive filler loading disrupts the polymer chain alignment and reduces crystallinity, possibly due to the agglomeration of NiCr particles and the restriction of chain motion. Additionally, the decrease in enthalpy associated with melting further supports the decline in overall crystallinity.

Overall, the DSC results indicate that the incorporation of NiCr affects the thermal stability and crystallization behavior of PA66. Low NiCr content may enhance crystallinity and thermal performance, while high filler loadings interfere with polymer ordering, leading to a more amorphous structure and altered melting characteristics.

These thermal findings, in combination with FTIR and conductivity data, demonstrate that NiCr serves a dual role in enhancing thermal conductivity and modulating the crystalline structure, thereby influencing the thermal and mechanical performance of the PA66 matrix.

**Figure 7:**
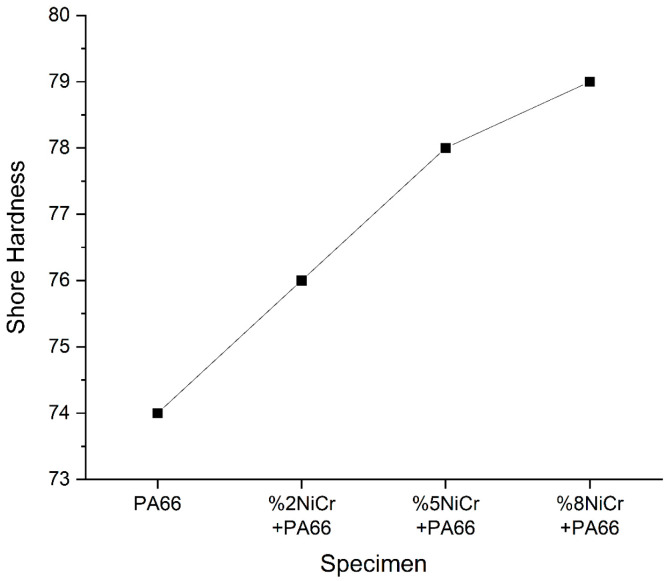
Shore hardness values of neat PA66 and PA66/NiCr composites at different filler loadings.

The Shore hardness of neat PA66 and NiCr-reinforced PA66 composites were measured to evaluate the effect of NiCr filler content on the mechanical performance, particularly surface rigidity and resistance to deformation. As presented in Figure 7, the incorporation of NiCr micro/nano particles into the PA66 matrix leads to a notable enhancement in hardness.

Neat PA66 exhibited a Shore hardness value of approximately 74, which is characteristic of its semi-crystalline and moderately flexible nature. Upon the addition of 2 wt% NiCr, the hardness increased to 76, indicating that the filler restricts polymer chain mobility and contributes to load-bearing capacity. With 5 wt% NiCr, the hardness value further rose to 78, and reached approximately 79.5 at 8 wt% NiCr.

The progressive increase in hardness is attributed to the reinforcing effect of NiCr particles, which act as rigid fillers within the polymer matrix. These particles limit the movement of polymer chains, enhance interfacial interactions, and improve the composite’s ability to resist surface indentation. Additionally, the uniform dispersion of NiCr throughout the matrix contributes to improved stress distribution under loading.

These findings demonstrate that NiCr significantly enhances the mechanical rigidity of PA66, making the composites more suitable for applications that demand high surface hardness, wear resistance, and dimensional stability under mechanical stress.

This improvement in hardness aligns with DSC results indicating modified crystallinity and supports the dual role of NiCr in enhancing both thermal and mechanical properties of PA66-based composites.

**Figure 8:**
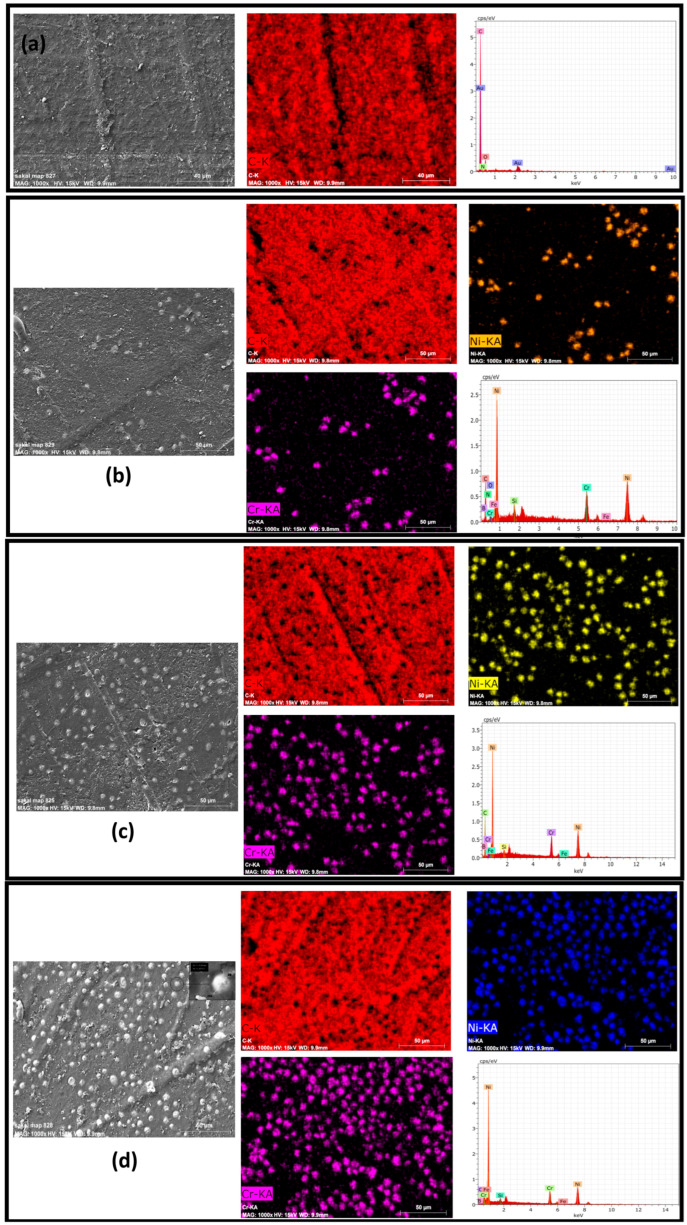
SEM images and EDS elemental maps of (**a**) pure PA66, (**b**) 2 wt% NiCr, (**c**) 5 wt% NiCr, and (**d**) 8 wt% NiCr composites.

The surface morphology and elemental composition of the PA66 matrix and NiCr-reinforced PA66 composites were examined using Scanning Electron Microscopy (SEM) and Energy Dispersive X-ray Spectroscopy (EDS). Representative micrographs and corresponding EDS elemental maps are presented in Figure 8, for:

(a) pure PA66, (b) 2 wt% NiCr + PA66, (c) 5 wt% NiCr + PA66, and (d) 8 wt% NiCr + PA66.

In the pure PA66 sample (a), the SEM micrograph shows a relatively smooth and homogeneous surface without the presence of filler particles. The corresponding EDS spectrum confirms the dominant presence of carbon (C) and oxygen (O), characteristic of the polymer matrix.

Upon addition of 2 wt% NiCr (b), the SEM image reveals the initial appearance of dispersed particles, with the EDS maps clearly identifying nickel (Ni) and chromium (Cr) distributions. The elemental maps show good dispersion with minimal clustering, and the EDS spectrum confirms the presence of Ni and Cr peaks along with C and O.

In the 5 wt% NiCr composite (c), the density of metallic particles increases, as shown in the SEM micrograph. EDS elemental maps illustrate a more pronounced and homogeneous distribution of Ni and Cr, indicating effective incorporation of the filler into the matrix. However, localized regions with slightly higher concentration suggest early stages of agglomeration.

For the 8 wt% NiCr composite (d), SEM imaging reveals a higher population of particles with occasional particle agglomerates, especially at the microscale. The Ni and Cr elemental maps remain well-distributed but with visibly denser clusters. The EDS spectrum confirms stronger Ni and Cr peaks, validating the increased filler content.

Overall, SEM–EDS analyses confirm that NiCr particles are successfully embedded within the PA66 matrix and evenly dispersed at low to moderate contents. However, at higher loadings, agglomeration tendencies increase, potentially affecting mechanical properties and interfacial bonding.

## 4. Discussion

The incorporation of waste micro–nano NiCr (80/20) powders into a PA66 matrix has demonstrated a multifaceted impact on the composite’s mechanical, thermal, electrical, and microstructural characteristics. These effects can be comprehensively interpreted in the light of existing literature on metal–polymer composites and the working hypotheses established at the outset of this study.

### 4.1. Mechanical Property Enhancement and Ductility Trade-Off

The tensile test results indicate that NiCr reinforcement improves both tensile modulus and tensile strength, with maximum gains observed at 8 wt% filler content. This aligns with earlier studies where rigid metallic fillers acted as stress-bearing entities, improving load transfer and stiffness due to strong interfacial interactions between filler and polymer matrix. The high intrinsic modulus of NiCr, coupled with good particle dispersion (as evidenced by SEM–EDS at lower loadings), likely facilitates efficient stress distribution.

However, a clear reduction in elongation at break was observed, which is consistent with the common ductility–strength trade-off in metal-filled thermoplastics. The reduced polymer chain mobility, confirmed by FTIR peak attenuation and broadening, suggests that NiCr particles restrict segmental motion, resulting in more brittle fracture behavior. This phenomenon has been reported for other PA66 composites reinforced with ceramic or metallic fillers, where improved stiffness comes at the expense of deformability.

### 4.2. Hardness and Wear Resistance Implications

The progressive increase in Shore D hardness with filler content reflects the rigid-filler effect, where metallic inclusions hinder surface deformation. This enhancement is advantageous for wear resistance which is critical in tribological applications. The observed hardness trend is in agreement with studies on metal–polyamide hybrids, where hardness improvements correlate with filler dispersion quality and interfacial bonding strength.

### 4.3. Thermal Conductivity and Heat Management Potential

The significant improvement in thermal conductivity, from 0.29 W/m·K (neat PA66) to 0.38 W/m·K (8 wt% NiCr), underscores the role of NiCr as an efficient heat conduction pathway. This is consistent with percolation-based thermal transport theories, where increased particle–particle contact promotes phonon/electron transfer. Given that NiCr exhibits high intrinsic thermal conductivity, the formation of partial conductive networks within the PA66 matrix at higher loadings is plausible. These improvements open the possibility for PA66/NiCr composites to be used in thermal interface materials, heat sinks, or electronic housings which requires enhanced thermal dissipation.

### 4.4. Electrical Conductivity Evolution and Percolation Behavior

While absolute electrical conductivity values remained in the insulation range, the increasing trend with NiCr content indicates proximity to an electrical percolation threshold. At low loadings (2 wt%), filler particles are insufficiently connected, whereas at 8 wt% the improved networking facilitates limited electron transport. Given σ ≈ 10^−12^ S/cm, the composites remain insulating; thus stand-alone EMI shielding is not expected at ≤8 wt% NiCr. Achieving meaningful SE typically requires orders-of-magnitude higher σ and/or hybrid conductive networks (e.g., carbon black/MWCNT + metal), which falls outside the present scope.

### 4.5. Thermal Transitions and Crystallinity Modulation

DSC results revealed a particular effect of NiCr loading on PA66 crystallinity. At low content (2 wt%), NiCr appears to act as a nucleating agent, slightly increasing melting temperature and possibly crystallinity. At higher content (8 wt%), the reduction in peak intensity and broadening suggest disruption of crystalline ordering, likely due to agglomeration and steric hindrance. Quantitative analysis confirmed this trend: Xc (neat) = 31.9%, Xc (2 wt%) = 33.7%, Xc (5 wt%) = 32.5%, and Xc (8 wt%) = 30.1%. Thus, while low NiCr loading promotes nucleation and crystallinity, higher contents reduce crystalline ordering. This dual role—crystallinity enhancement at low content and disruption at high content—parallel trends seen in other nanoparticle-reinforced polymers, where particle dispersion and interfacial adhesion dictate the crystallization outcome.

The observed stiffness and hardness increase is comparable to PA66–Al_2_O_3_ microcomposites [16], and lower than PA66–carbon black/glass fiber hybrids [15]. Unlike carbon fillers, NiCr requires higher loading for electrical effects but improves thermal conductivity and hardness.

### 4.6. Microstructural Insights and Agglomeration Effects

SEM–EDS mapping confirmed that good particle dispersion is achievable at lower NiCr contents, while higher loadings (8 wt%) lead to localized agglomeration. This aggregation can generate stress concentration sites and reduce the reinforcing efficiency, which could explain why mechanical performance gains do not proportionally match the filler increase beyond certain thresholds. Controlling dispersion—possibly through surface modification of NiCr particles or coupling agents—could mitigate this limitation in future work. No coupling agent was employed in this study; future work may explore the use of surface modification or coupling strategies to further improve particle dispersion and interfacial adhesion.

### 4.7. Sustainability and Industrial Relevance

From a sustainability perspective, the effective reuse of waste NiCr powders from the coatings industry highlights a circular economy pathway, converting industrial by-products into high-value composite reinforcements. This not only addresses waste management concerns but also reduces the reliance on virgin raw materials, aligning with current global trends in sustainable materials engineering.

In conclusion, the results demonstrate that waste-derived micro–nano NiCr can be an effective reinforcing agent for PA66, providing simultaneous mechanical, thermal, and functional property enhancements, while supporting sustainable materials development.

## 5. Conclusions

This study demonstrated that incorporating waste-derived micro–nano NiCr (80/20) powders into a PA66 matrix can significantly enhance the composite’s mechanical, thermal, and functional properties while promoting sustainable material use. Tensile modulus, tensile strength, hardness, and thermal conductivity all increased progressively with NiCr loading, with the highest values obtained at 8 wt% filler content. These improvements are attributed to the high stiffness and intrinsic conductivity of NiCr, efficient load transfer, and partial formation of conductive networks within the polymer matrix.

However, the enhancement in strength and rigidity was accompanied by a reduction in elongation at break, reflecting the typical ductility–stiffness trade-off observed in rigid-filler composites. DSC and FTIR analyses revealed that low NiCr content can promote nucleation and crystallinity, while higher loadings tend to disrupt crystalline domains due to particle agglomeration and restricted chain mobility. DSC-derived crystallinity analysis indicated nucleation at low NiCr content and crystallinity disruption at 8 wt%, consistent with the observed thermal and mechanical behavior. SEM–EDS observations confirmed uniform filler dispersion at lower contents and increasing agglomeration at higher loadings.

From a functional perspective, thermal conductivity improvements and the observed trend toward electrical conductivity percolation suggest potential applications in heat dissipation, antistatic components, and electronic housings. Importantly, using industrial waste NiCr powder as a reinforcement not only offers performance gains but also supports circular economy principles by valorizing by-products from the coatings industry.

Taking all into consideration, the findings indicate that waste NiCr/PA66 composites are promising candidates for high-performance, environment friendly applications in automotive, electronics, and thermal management systems. Future work should focus on optimizing filler dispersion and content to balance mechanical performance with ductility, as well as exploring hybrid reinforcement strategies for multifunctional composites.

Valorizing waste NiCr powders avoids landfilling or costly recycling, with potential waste-handling cost savings of ~200–300 €/ton. Thus, this approach provides both performance gains and ecological benefits.

## Figures and Tables

**Table 1:** Crystallinity values.

Specimen	NiCr (wt%)	ΔH_m_ (J/g) *	X_c_ (%)
PA66 (neat)	0	60	31.9
PA66 + NiCr (2%)	2	62	33.7
PA66 + NiCr (5%)	5	58	32.5
PA66 + NiCr (8%)	8	52	30.1

## Data Availability

The original contributions presented in this study are included in this article. Further inquiries can be directed to the corresponding author.
